# Are Australian prisons meeting the needs of Indigenous offenders?

**DOI:** 10.1186/s40352-016-0045-7

**Published:** 2016-12-06

**Authors:** Stephane M. Shepherd, James R. P. Ogloff, Stuart D. M. Thomas

**Affiliations:** 1 Centre for Forensic Behavioural Science, Swinburne University of Technology and Forensicare, 505 Hoddle Street, Clifton Hill, Victoria 3000 Australia; 2 Justice and Legal Studies, School of Global, Urban and Social Studies, RMIT University, Melbourne, Victoria 3000 Australia

**Keywords:** Prisoners, Correctional Health care, Indigenous offenders, Risk needs, Psychological distress, Violence

## Abstract

**Background:**

The over-representation of Indigenous Australians in custody is well documented, yet little is known about whether the health and social needs of Indigenous prisoners are met in correctional facilities. This study sought to identify common areas of need in a representative sample of Indigenous people in custody, and consider how well prison services were addressing these issues.

**Methods:**

The sample comprised 122 Aboriginal and Torres Strait Islander people in custody in Victoria. Participants were administered the Camberwell Assessment of Need Forensic-Short Version to ascertain the presence or absence of needs in custody. Statistical analyses to determine associations with re-offence were conducted.

**Results:**

Findings indicated that prisons were able to meet the non-criminogenic needs of many offenders; however there was a limited capacity to address specific criminogenic needs. Psychological distress, substance abuse, poor treatment adherence and threatening behaviours were considered ongoing needs regardless of supports/interventions being provided. Moreover, these four unaddressed needs were all associated with future recidivism.

**Conclusions:**

Effective prison treatment services focusing on these four areas of need are urgently required. Such initiatives require continuation post-release combined with additional assistance to uphold basic non-criminogenic needs acquired in prison.

## Background

The mass incarceration of Indigenous Australians is an enduring concern. Indigenous people are disproportionally imprisoned in every Australian state and territory (Australian Bureau of Statistics [ABS] 2015). This matter receives ongoing publicity in Australia, including recent appeals to set national reduction targets on Indigenous prison rates. The numbers of Indigenous prisoners continue to climb despite widespread political attention and a recent decline in non-Indigenous youth imprisonment figures (Australian Institute of Health and Welfare [AIHW] 2015a).

The factors underpinning Indigenous contact with the criminal justice system are becoming clearer. Representative surveys of the Indigenous population identify several key factors (criminogenic needs) that increase the likelihood of arrest. These include substance abuse, low educational attainment, unemployment, exposure/experience of violence and prior contact with the justice system (Ferrante 2013; Weatherburn et al. 2006, 2008). Surveys of Indigenous prisoners add further weight to the commonality of these risk factors (AIHW 2015b; Heffernan et al. 2012; Indig et al. 2010, 2011; Lawrie 2003). Though few in number, investigations identifying which of these risk items predict recidivism for Indigenous offenders find that prior criminal justice system involvement and socio-environmental markers predominate (Shepherd et al. 2014; Wundersitz 2010). While these findings are instructive, the Royal Commission into Aboriginal Deaths in Custody (RCIADC) report highlighted similar concerns back in 1991 outlining solutions to both reduce Indigenous deaths in custody and high imprisonment rates (RCIADC 1991). Although many of the recommendations have not been meaningfully implemented (Amnesty International Australia 2015), some have materialised in various forms, including cultural awareness training for both police and correctional officers and the hiring of Indigenous workers in justice settings to provide cultural assistance and culturally relevant program delivery. Common antecedents to criminal activity are placed within broader contemporary and historical societal structures contributing to present day inequities. As such, Indigenous holistic frameworks of health and healing comprise the retrieval and exercising of cultural processes to offset injustices prompted by colonialism (Hovane et al. 2014).

Given that regional governments have increased efforts to acknowledge the utility of alternative therapeutic models for Indigenous prisoners, there is a growing community expectation that problem areas for Indigenous prisoners will be readily addressed. The most recent national incarceration figures, however, suggest otherwise. The reasons for this latest setback perhaps include, at best, a continued institutional inability to grasp or underestimate the complexity of the problem and at worst, indifference to the problem. Conversely, it may simply represent an institutional aversion to employing a series of what has been described as straightforward recommendations deemed to be ostensibly burdensome (Shepherd and Phillips 2016). Either way, a re-visiting of the needs of Indigenous prisoners represents a practical starting point for both gauging the existing correctional accommodation of needs and identifying potential areas for institutional improvements.

Key issues underpinning a swift return to prison for Indigenous people include release into the community with numerous unsupported health, basic living, social and therapeutic needs. Moreover, addressing needs in prison settings is less impactful if supports and interventions are not sustained post-release. In this scenario, a met need can easily devolve into an unmet need. Nonetheless, surveying which health and social problems prison services are currently able to address or not address, for Indigenous prisoners enlightens prospective attempts to improve service provision as well as potentially reduce recidivism rates (Cutcher et al. 2014; Kariminia et al. 2007, 2012). That is the focus of this study.

The aims of the study are to identify common areas of need for adult Indigenous Australian people in custodial settings. This includes the total number of needs, needs which have been or are being met within correctional settings, and needs which remain unmet. We also aim to identify which combination of needs contributed to an individual’s index offence. Through tracking participants for 24 months post-release, we seek to determine associations between need and re-offending.

Given the continued high rates of Indigenous incarceration, we hypothesize that participants will have elevated levels of aggregate need. We also hypothesize that participants with higher levels of unmet need will re-offend earlier.

## Method

### Participant details

All remanded and sentenced Aboriginal and Torres Strait Islander prisoners from 11 regional and metropolitan prisons in the state of Victoria were approached to participate in the study. Participants were required to have their Aboriginal and Torres Strait Islander status formally registered with prison services. There was no recruitment from one correctional centre due to insufficient numbers of Indigenous prisoners. A total of 122 participants (male = 107; female = 15) was recruited. The sample size is representative of Aboriginal and Torres Strait Islander prisoners in Victoria prisons (8%); proportionally, the smallest of any state in Australia (ABS 2015).

The mean age of the sample was 34.4 (*SD* = 10.3, *range* = 19–63) years. The majority of participants were born in Victoria (61.1%, *N* = 69), and approximately 20% (*N* = 32) of participants were born in New South Wales. The majority of participants identified as Yorta Yorta or Gunai. Most participants had not completed high school (male: 90.6%, female: 93.3%). The majority (77.7%, *N* = 94) listed Centrelink (government assistance payments) as their main source of income and only 15% (*N* = 18) indicated having full-time employment prior to custody. The majority of participants were sentenced at the time of the interview with fewer than 30% on remand. Violent offences were the most prominent index offences for males (72.6%) and females (46.7%). The mean current sentence being served was 30.8 months. Males had significantly longer current sentences (*t* = 4.33, *p* < 0.001).

### Procedure

The data presented in this report were based on an interviewer-administered needs questionnaire. Aboriginal Wellbeing/Liaison Officers at each prison briefly informed participants of the details of the study. Those prisoners interested in participating in the study then met with the interviewers who provided them with an explanatory statement. Interviews were conducted by two interviewers, one of whom was a culturally trained mental health clinician and the other an Aboriginal and Torres Strait Islander research officer. At the commencement of the interview, the Aboriginal and Torres Strait Islander research officer verbally reviewed the explanatory statement with the prisoner and provided an opportunity for the prisoner to ask questions. Prisoners who wished to take part were asked to sign a consent form acknowledging their understanding of the study. All interviews were conducted in private rooms visible (though not audible) to custodial staff. Participation in the study was voluntary and participants could choose not to answer any questions, or terminate the interview at any time, if desired.

### Instrument

The Camberwell Assessment of Need Forensic-Short Version (CANFOR-S, Thomas et al. 2003, 2008) is a validated assessment instrument designed to identify the needs of forensic mental health service users across 25 different domains.

Through a series of structured questions, an assessor determined the presence or non-presence of client needs. A need is defined as being present if a client has had prior complications in that particular area in the past month. A met need is defined as a problem area for a client that is currently being addressed through existing prison services, or other informal help. An unmet need is defined as a problem area where no interventions/services are currently being provided and/or existing interventions/services are ineffective. The total need score is defined as the sum of the number of met needs and unmet needs. Items were recorded by clinicians as follows; 0 = No problem, 1 = Met need, 2 = unmet need. Sixteen of the items are additionally considered dichotomously (0 = No, 1 = Yes) in relation to their contribution to the index offence. Project consultants decided that the CANFOR-S item ‘sexual expression’ would not be obtained during the study given that both assessors were female. As such, 24 of the 25 CANFOR-S need domains were considered in this study.

Previous studies have administered the CANFOR-S with samples of forensic psychiatric patients and gaol detainees (Baksheev et al. 2010; Castelletti et al. 2015; O’Hara et al. 2016; Segal et al. 2010). While rarely used in prison samples, the range of needs it assesses is clearly relevant to prisoners in addition to forensic patients (Thomas et al. 2009).

#### Recidivism

Criminal histories from the Victoria Police Law Enforcement Assistance Program (LEAP) database were obtained for all consenting participants for 2 years post custodial interview. The LEAP database records all contacts people in Victoria have with the Victoria Police, both as offenders and victims. Recidivism is defined as any police charge post assessment. Violent crimes are acts intended to cause or threaten to cause physical harm. General crimes encompass all charges.

### Data analyses

Preliminary descriptive statistics were conducted to determine both the prevalence of each CANFOR item by met/unmet status and the mean number of met/unmet needs for the entire sample. Next, the extent of recidivism was ascertained for: i) all participants who were released during the follow up period; ii) released participants whose number of unmet needs exceeded the sample median ii) released participants whose number of total needs exceeded the sample median. Subsequent chi-squared tests of association and survival analyses (Kaplan-Meier) were employed to detect significant group differences across levels of need for recidivistic outcomes. Receiver Operating Characteristic (ROC) analyses were then employed to determine whether recidivists were more likely to receive higher numbers of total needs/unmet needs compared to non-recidivists. Chi-squared tests of association and Mann–Whitney U tests were both utilised to identify differences in need according to whether a participant re-offended or not. Due to the number of comparisons here, alpha was adjusted to *p* = 0.003. Finally, logistic regression was employed to determine which CANFOR items were predictive of general and violent recidivism.

## Results

### Individual needs

The presence of different need domains across the sample are summarised in Table 1. The most common areas of need included Daytime activities, Child care, Money, Information about condition/treatment, Food, Company and Psychological distress. The bulk of the areas of need identified were considered *met* for the majority of participants. Only the Food, Psychological distress and Safety to others items remained *unmet* for greater proportions of the sample. Items most frequently reported as contributing to a participant’s index offence included Drug use, Psychological distress, Alcohol use and Safety to others.

**Table 1:** Presence of needs across CANFOR domains

	Area of need	Area of need			Contribution to index offence (%)
	*N* (%)	Met (%)	Unmet (%)	Met/Unmet (%) + -	
Accommodation (*N* = 81)	45 (55.6)	59.9	39.9	+20.0	27.0
Food (*N* = 116)	76 (65.5)	36.8	63.2	−26.4	N/A
Looking after the living environment (*N* = 119)	46 (38.7)	84.8	15.2	+69.6	N/A
Self-care (*N* = 119)	41 (34.5)	95.1	4.9	+90.2	N/A
Daytime activities (*N* = 119)	99 (83.2)	58.5	41.5	+17.0	43.9
Physical health (*N* = 119)	65 (54.6)	60.1	39.9	+20.2	N/A
Psychotic symptoms (*N* = 118)	30 (25.4)	60.2	39.8	+20.4	23.9
Information about condition and treatment (*N* = 110)	75 (68.2)	60.0	40.0	+20.0	N/A
Psychological distress (*N* = 119)	75 (63.0)	29.4	70.6	−41.2	55.3
Safety to self (*N* = 119)	28 (23.5)	64.3	35.7	+28.6	22.6
Safety to others (excluding sexual offences and arson) (*N* = 116)	38 (32.8)	39.3	60.7	−21.4	50.4
Alcohol (*N* = 87)	14 (16.1)	57.1	42.9	+14.2	51.8
Drugs (including solvents) (*N* = 88)	33 (37.5)	63.7	36.3	+27.4	62.3
Company (*N* = 117)	75 (64.1)	70.7	29.3	+41.4	46.5
Intimate relationships (*N* = 103)	55 (53.4)	50.9	49.1	+1.8	37.4
Child care (*N* = 86)	77 (89.5)	64.9	35.1	+29.8	24.0
Basic education (*N* = 117)	60 (51.3)	61.1	38.9	+22.2	N/A
Telephone (*N* = 118)	63 (53.4)	85.8	14.2	+71.6	N/A
Transport (*N* = 45)	4 (8.9)	75.3	24.7	+50.6	N/A
Money (*N* = 118)	60 (50.8)	70.1	29.9	+40.2	40.0
Benefits (*N* = 85)	38 (44.7)	55.3	44.7	+10.6	14.3
Treatment (*N* = 93)	75 (80.6)	65.4	34.6	+30.8	20.4
Sexual offences (*N* = 24)	9 (37.5)	88.8	11.2	+77.6	34.8
Arson (*N* = 18)	3 (16.7)	100.0	0.0	+100.00	18.8

Note: Areas of need derived from the CANFOR instrument

The mean number of met, unmet, index offence and total needs for the overall sample are summarised in Table 2. Participants presented with almost 10 total needs (met needs + unmet needs) on average. The mean number of met needs exceeded the mean number of unmet needs. An average of five different need domains contributed to participants’ index offences. No significant differences were detected across gender.

**Table 2:** Mean sample needs

	*M* (*SD*)	Range	95% CI
Met needs	5.86 (3.47)	0–15	5.19–6.48
Unmet needs	3.99 (3.22)	0–14	3.39–4.60
Total needs	9.75 (4.18)	0–22	9.00–10.50
Index offence needs	5.11 (3.21)	0–14	4.52–5.71

Note: *N* = 118; Needs derived from the CANFOR instrument

### Follow-up analyses

#### Re-offending and time at risk

Thirty eight participants had not been released from custody throughout the duration of the follow up period, so the eligible sample was reduced to 84 participants. Released participants were significantly younger than participants who were not released (*t* = −3.16, *p* < 0.01). Participants who were not released were undertaking longer custodial sentences (*t* = 3.21, *p* < 0.01) and had spent significantly more timein prison as an adult compared to released participants (*t* = 2.83, *p* < 0.01). No significant differences were found across the number of previous custodial episodes by release status (*t* = 0.81, *p* = 0.42). During the follow up period, 56% (*N* = 47) of participants released from custody reoffended (see Table 3). Across gender, 58.1% of males re-offended and 50% of females re-offended. More than 40% (*N* = 34) of released participants were charged with a violent offence.

**Table 3:** Group re-offending and survival

	GR	Time (Months) to GR (*M*, *SE*)	VR	Time (Months) to VR (*M*, *SD*)
Total Sample	56.00%	13.54 (1.35)	40.50%	17.27 (1.13)
Unmet Needs (4+)	69.77%	10.26 (1.86)	53.49%	14.94 (1.75)
Total Needs (10+)	65.96%	12.38 (1.79)	44.68%	16.77 (1.50)

*N* = 84; *GR* General Re-offence, *VR* Violent Re-offence

Almost 70% (*N* = 59) of participants who presented with 4 (sample median cutoff) or more unmet needs reoffended with 53.49% (*N* = 45) reoffending violently. Participants with four or more unmet met needs were more than three times more likely to reoffend compared to participants with fewer than four unmet needs (*χ* ^2^(1) = 6.82, *p* = 0.01). Participants with four or more unmet needs were also more likely to reoffend, both generally (χ^2^_log_(1) = 6.49, *p* = 0.01) and violently (χ^2^_log_(1) = 3.53, *p* = 0.06), earlier compared to participants with fewer than four unmet needs.

For participants who recorded 10 or more total needs, almost two-thirds reoffended and over 44% violently reoffended. Those with 10 or more total needs were 2.5 times more likely to reoffend compared to participants with fewer than 10 total needs (*χ* ^2^(1) = 4.33, *p* = 0.04). No differences were detected for survival times across total need groups (Table 4).

**Table 4:** Instrument discrimination

	General recidivism		Violent recidivism	
	*R*	AUC (*SE*)	*R*	AUC (*SE*)
Unmet needs	0.21*	0.64* (0.06)	0.15	0.60 (0.06)
Total needs	0.14	0.59 (0.06)	−0.02	0.49 (0.07)

Note: (*N* = 84)

*p < .05

Areas under the curve were performed to determine the discriminative utility of CANFOR domains (see Table 4). Only the unmet needs domain reached significance for general recidivism. This means that a randomly selected general recidivist is a 64% chance of presenting with a greater number of unmet needs compared to a randomly selected general non-recidivist. The number of unmet needs was also significantly correlated with general recidivism, though this was weak in magnitude. The number of total needs did not meaningfully discriminate according to the general or violent recidivism category.

#### Levels of need by recidivism status

Recidivists (*M* = 4.57, *SD* = 2.00) had significantly higher levels of unmet needs compared to non-recidivists (*M* = 3.43, *SD* = 3.11; *U* = 626.00, *p* = 0.03). For recidivists, the most common unmet areas of need included: Psychological distress (47.9%), Food (47.9%), Daytime activities (39.6%), Treatment (38.9%) and Child care (35.0%). Specifically, recidivists were 2.8 times more likely than non-recidivists to have ‘treatment’ needs unmet however this effect did not reach significance (*χ* ^2^(1) = 3.04, *p* = 0.08).

The most common ‘met’ needs for recidivists included: Telephone use and accessibility (59.6%), child care (57.5%), company (52.2%), information about medical condition and treatment (41.9%) and agreement with treatment prescribed (41.7%). Recidivists however, were almost three times less likely to have the treatment item as a ‘met’ need compared to non-recidivists although this finding was non-significant (*χ* ^2^(1) = 3.87, *p* = 0.05). No meaningful differences were detected across levels of total met needs by recidivism status.

### Predictors of re-offending

CANFOR ‘index offence’ items that reached a prevalence of more than 40% of the sample were included in a stepwise logistical regression analyses to identify the strongest predictors of re-offending. The item ‘treatment’ was also included as a covariate due to its significance in previous analyses. Items in the initial model comprised: Daytime activities, Psychological stress, Safety to others, Alcohol, Drugs, Company, Money, and Treatment. A correlation matrix of predictors discovered no significant bivariate correlations greater than *r* = 0.25.

No significant prediction model was obtained for general recidivism. For violent recidivism, a prediction model featuring the item ‘Drugs’ was reached in one step (*χ* ^2^(1) = 4.87, *p* = 0.03). The Hosmer Lemeshow test statistic did not reach significance (*χ* ^2^(1) = 0.28, *p* = 0.60) indicating an acceptable model fit. Presenting with drug use as an area of need significantly predicted violent recidivism (*Exp* (B) = 2.55, *p* = 0.04).

## Discussion

This study aimed to identify areas of need and their relationship with re-offending for a representative cohort of Indigenous Australians in custody. Findings indicated that while correctional facilities were generally able to address numerous areas of need, many participants had elevated areas of need, with several remaining unaddressed and connected to subsequent recidivism.

### Prevalence of need

#### Pre-existing areas of need

Recurring prior areas of need identified in the sample were spread across clinical, personal and socio-economic domains. Participants consistently reported having ‘little to do’ throughout the day and being unhappy with their social lives. The majority of participants also experienced psychological distress. Elevated levels of mental illness and psychological distress have been found among Australian Indigenous people in both correctional settings and in the general population (Butler et al. 2007; Dudgeon et al. 2014; Heffernan et al. 2012; Jorm et al. 2012; (Ogloff, Pfeifer, Shepherd, & Ciorciari: Assessing the mental health, substance abuse, cognitive functioning and social/emotional well-being needs of Aboriginal prisoners in Australia, submitted). There appeared to be a discrepancy between participants views of their needs for treatment and those being prescribed/provided. This may have occurred because of a lack of cultural responsive service provision, poor clinician-patient cross-cultural communication, a mistrust of Western clinicians/medicine, or an inability to accommodate Indigenous models of health and holistic therapy which can include spiritual concepts. Relevant to this point, participants commonly reported receiving unclear information about their current medications, replicating prior research (Carroll et al. 2014). Another prevalent area of need identified for participants was limited contact/access to their children.

#### Met/Unmet

Prison services demonstrated a capacity to address participant areas of need to a moderate extent. For the majority of the prominent areas of concern described above, more prisoners had their needs met within the correctional environment than those who did not. This situation improved in prison for over 70% of participants who were previously unhappy with their social life and limited contact with others.. Relatedly, over 60% of participants reported involvement in meaningful daytime activities. Some improvement was also perceived by participants regarding treatment. Two-thirds of those who were initially unhappy with their treatment plan were now in agreement with treatment/medication prescribed for them. Similarly higher proportions of participants who earlier flagged inadequate treatment interaction, now reported improved communication in this area. Of the highly prevalent areas of concern initially outlined, two remained unaddressed for the majority of participants. Less than one-third of participants reported custodial assuagement of their psychological distress. High rates of depressive episodes, anxiety and post-traumatic stress disorder were recently observed in samples of Indigenous prisoners (Heffernan et al. 2012, 2015; (Ogloff, Pfeifer, Shepherd, & Ciorciari: Assessing the mental health, substance abuse, cognitive functioning and social/emotional well-being needs of Aboriginal prisoners in Australia, submitted). Whether elevated rates of psychological distress are the result of social adversity prior to custody or the experience of custody itself (or a combination of the two), Australian correctional facilities have long struggled with providing appropriate and effective services in this space. The ability to prepare one’s food was another outstanding unaddressed area of need; however, this issue is most likely a reflection of custodial security concerns and communal food distribution.

Other areas of need (though less prevalent) were *met* in prison for the majority of participants. Almost all prisoners who previously had difficulties with self-care were now able to physically take better care of themselves. This maintenance extended to participants’ living environment. For participants who had initial outstanding physical health concerns, over 60% were now feeling better physically. Furthermore, approximately 60% of participants who reported prior alcohol and drug problems no longer exhibited these difficulties in custody. Whether this improvement is likely to be the result of diminished alcohol and drug access in custody, or the undertaking of beneficial substance abuse treatment in custody is unknown. Lowered drug and alcohol usage may have also contributed to reported improvements in physical health and self-care. Also, psychotic symptoms appeared to diminish for a majority of participants who reported this as a prior area of need. This may be a product of these needs being identified and then the person a potential function of receiving custodial treatment and monitoring. The same phenomenon was observed for risk of self-harm. For participants reporting previous issues with reading and writing, over 60% noted improvement in this area. As such, basic education programs conducted within custodial settings appear to be useful for some Indigenous prisoners denoting a meaningful daytime activity. Money problems, paying bills and access to a telephone were also issues alleviated through incarceration. Of note, participants previously at risk for sex and/or arson offences appeared satisfied with the help they were receiving in custody for these problems. These specific participants however were a minority within the overall sample and thus caution is required when generalising such findings. A final area of need for some participants that was noticeably *unmet* was threatening behaviour and controlling one’s temper (Safety to others). For more than 60% of participants who identified this as a problem, help being received was either considered to be unavailable or ineffective.

Items commonly recorded as influential to participants’ index offences included Drug abuse, Alcohol use, Psychological distress and Safety to others. This finding is perhaps unsurprising given that almost three-quarters of the sample were currently imprisoned for a violent offence, broadly defined. Of concern, however, is that two of the above items were considered *unmet* for a majority of participants in Psychological distress and Safety to others. Prior research indicates that high levels of psychological distress often persist after release from custody (Thomas et al. 2016). Further, although a slight majority of participants were receiving assistance for substance abuse problems, a significant minority of participants’ needs were considered to be unmet here. In prior research Indigenous prisoners reported higher levels of alcohol consumption compared to non-Indigenous offenders in the period leading to incarceration (Kinner et al. 2012). So, in effect, it appears that four (likely interrelated) items most associated with past offending remained predominantly unmet in correctional settings.

### Collective areas of need

Participants presented with approximately ten areas of need on average. This was significantly higher than previous Australian studies in other correctional/forensic settings (Baksheev et al. 2010; Segal et al. 2010) and Australian studies featuring Indigenous populations (Davison, et al. 2015). While previous studies also discovered psychological distress and substance use to be significant areas of prisoner/patient need, the ‘safety to others’ item was less prominent compared to the current study. This finding aligns with the high rate of violent index offences observed among study participants. On average, participants had a marginally higher number of met needs compared to unmet needs. Although this is an indication that the prison environment is addressing more than half the overall needs of Indigenous prisoners, it is likely that most of these *met* needs are not centrally related to the likelihood of engaging in offending.

### Re-offending

Higher proportions of participants with four or more unmet needs re-offended compared to those with fewer than four unmet needs. Moreover, possessing more than four unmet needs was associated with a shorter time to re-offence, for both general and violent offences. Having a greater number of needs overall was also associated with recidivism though to a lesser extent and did not affect participant time at risk. These findings indicate that when it comes to recidivism the general accumulation of different areas of need, though important, is perhaps less impactful than *which* specific areas of need remain unaddressed.

Investigating the met and unmet needs of re-offenders produced several interesting results. For many recidivists, high levels of psychological distress were considered to be unaddressed when they were in prison. Similarly, participation in meaningful daytime activities and agreement with treatment prescribed were unfulfilled requirements for numerous re-offenders. Not adhering to medication may have prolonged psychological distress and prompted other problem behaviours (including institutional infractions) for prisoners who may have mental disorders or other sub-clinical concerns. This may have prevented the extent of their participation in daytime programs or group activities due to segregation or being monitored in a specialised unit. Release back into the community following such circumstances is evidently problematic and may, in part, explain these findings.

Recidivists were found to have similar levels of total need compared to non-recidivists, but higher levels of unmet needs, confirming earlier suggestions that the *quantity* of needs is less pertinent as compared to which unaddressed needs subsist. For example, problematic drug use significantly predicted violent re-offending. Substance abuse is regularly linked to criminal behaviour for Aboriginal offenders (AIHW 2015b; Ferrante 2013; Indig et al. 2010) and post-release mortality (Forsyth et al. 2014). Findings suggest that substance abuse treatment in prison appears to not be meeting the needs of Indigenous prisoners. More targeted supports and a need for follow-up aftercare services are necessary to ameliorate likelihood of relapse when leaving prison.

### Limitations

Some caution is recommended when generalising the study’s findings to Indigenous people in custody in other regions within Australia. Other jurisdictions may also have varying laws that differentially impact Indigenous people and/or distinct correctional conditions and available treatment schemes. Assessors in the study also took into account mood states which can impact on the perceptions of met and unmet needs. Another limitation was the small number of female participants in the sample which precluded the provision of meaningful gender-specific aggregate data and analyses. It is possible that the risk profiles of female prisoners in the sample differ from those of male prisoners. Finally, the cross-sectional nature of the data only provided a snapshot of Indigenous prisoner needs at a particular moment in time. Needs in prison may vary depending on the amount of time served. Nonetheless, the study identified specific needs that appear to remain unaddressed prior to, during and post incarceration. Further research is needed to better understand which specific, or combination of, unaddressed needs prompt immediate recidivism after transitioning to the community from prison.

### Implications

The confining and disciplinary nature of correctional facilities is often at odds with notions of offender agency and rehabilitation. Findings here indicate, however, that prisons have the capacity to, and in some cases do successfully address many non-criminogenic needs of prisoners. Health improvements in custody for Indigenous people are noted in previous research (AIHW 2015b). Yet the ability to provide similarly effective solutions for specific criminogenic needs is considerably smaller. Four key areas of criminogenic need emerged in this study. Persistent psychological distress was a constant, pre, during and post-release for participants. Substance use (drugs and alcohol) was associated with both index and subsequent violent offending despite the correctional facilities reportedly meeting this need for many participants. Perhaps this points to ineffectual treatment or, more likely, a discontinuation of effective treatment post release. Correctional facilities that provide ‘one size fits all’ rehabilitation programs risk overlooking cultural specific methods that may be useful in mitigating distress and other problem behaviours. This has been noted previously when treating anger management issues with Indigenous prisoners (Day et al. 2008). Relatedly, anger/temper issues endangering others was the third key criminogenic need identified in this study. Finally, non-agreement/non-adherence with treatment was prominent among recidivists, although some recidivists were agreeable with prescribed treatment. Again, this is perhaps a combination of (culturally) ineffectual treatment and/or deficits with post-release supports and services. Furthermore, the non-criminogenic needs that are met in a prison environment may quickly become unmet in the community if the client returns to a dysfunctional setting. Here the life stability required to compliment treatment for criminogenic needs may be eradicated post-release. As such, support to conserve non-criminogenic needs fostered in prison should not be disregarded on return to the community.

## Conclusions

The met/unmet health and social needs of 122 Aboriginal and Torres Strait Islander people in custody were identified and then considered in relation to post-release recidivism. The general needs of the sample were predominantely met however four treatment related needs were unmet and related to future re-offence.

Greater efforts are required to provide accessible ongoing culturally meaningful treatment for Indigenous people in custody that extends beyond the prison environment. Treatment must be combined with strong health, legal and family support services to ensure its continuity and value.
